# Clinicopathologic characteristics and surgical outcome of synthetic fiber conjunctival granuloma

**DOI:** 10.1186/s12886-020-01717-1

**Published:** 2020-11-16

**Authors:** Zhang Chen, Tianyang Wang, Qintuo Pan, Zhaoliang Zhang, Zongduan Zhang

**Affiliations:** 1 Wenzhou Medical University Eye Hospital, 270 Xueyuan Road, Wenzhou, 325027 Zhejiang China; 2 Yantai Affiliated Hospital of Binzhou Medical University, Yantai, Shandong China

**Keywords:** Synthetic fiber conjunctival granuloma, Clinical feature, Histopathologic

## Abstract

**Background:**

Until recently, synthetic fiber conjunctival granuloma (SFCG) is rarely reported and has been poorly understood. Our study was to explore the clinical features, histopathologic characteristics, surgical outcomes, and prognosis of SFCG after surgical excision.

**Methods:**

Retrospective review of clinical findings, histopathological and immunohistochemical studies identified 18 cases of SFCG. Specimens were routinely processed and stained with H&E. Immunohistochemical stains for GMS, PAS, CD68 and CK-pan were also performed.

**Results:**

Eighteen patients with an average age of 9.3 ± 6.6 years had a tender white to red mass on the conjunctiva. All the lesions were completely removed, and none of the patients relapsed. Histologically, all of the specimens revealed inflammatory granulation tissues with a large number of inflammatory cells infiltration and the presence of synthetic fibers. Immunohistochemical stains were positive for CD68, CK, GMS and PAS.

**Conclusions:**

Synthetic fiber conjunctival granuloma is an uncommon lesion with foreign body sensation caused by inoculation of synthetic exogenous materials. These lesions are mostly unilateral and occur in the inferior conjunctival fornix. SFCGs are characterized by a large number of inflammatory cells infiltration and the presence of synthetic fibers. Surgical excision followed by topical corticosteroids has been clinically proven to be effective.

**Supplementary Information:**

The online version contains supplementary material available at 10.1186/s12886-020-01717-1.

## Background

Many non-synthetic and synthetic exogenous materials may play a vital role in causing foreign body conjunctival granulomas. To date, several studies have indicated synthetic fiber as a possible cause of conjunctival granuloma in children [1–3]. It is sometimes called “teddy bear” granuloma because some cases were caused by the materials used to produce stuffed animals [4]. It is also a rare granulomatous foreign body reaction of the conjunctiva [5]. The eye’s protective mechanism of blinking and tearing usually removes foreign bodies from the ocular surface. However, a research has reported that children were more tolerant to ocular irritation caused by foreign bodies, and foreign bodies may occasionally embed in the underlying stroma and be encapsulated by the mucous [6]. Retaining these materials may initiate a local chronic inflammatory response that cause lesions [4]. Synthetic fiber conjunctival granuloma (SFCG) is relatively rare. It is usually unilateral and located in the inferior conjunctival fornix. The patients usually have no history of trauma and are asymptomatic [5]. All of the lesions in our case series were excised surgically and sent for histopathological analysis. Microscopic examination revealed that the conjunctival granuloma was composed of granulation tissue with chronic inflammation and foreign body aggregates consistent with synthetic fibers. To date, very little research has been conducted on SFCG, and most studies have had small sample sizes. Thus, the clinical and histopathologic characteristics and treatment outcomes of the disease remain poorly understood. The present research with the largest case series of 18 patients aims to facilitate further understanding of the disease(An additional file shows this in more detail (see Additional file 1)).

## Methods

We retrospectively evaluated 18 patients who underwent surgical excision of SFCG between July 2011 and June 2019 at the Wenzhou Medical University Eye Hospital (Table 1). The primary selection criteria for the 18 patients was histologically proven SFCG. The medical records including the patients’ age and sex, clinical presentation, duration of signs and symptoms, location of mass, treatment modalities, and outcomes were retrospectively reviewed (Table 1). We also gathered clinical photographs obtained on eversion of the inferior eyelids and intraoperative findings of the patients. All of the patients underwent surgical excision by the same ophthalmologist. All of the conjunctival granulomas were excised completely under surface anesthesia except for one patient who underwent general anesthesia, followed by the administration of topical corticosteroid eye drops for 1 week. All of the surgical specimens were grossly examined for size and color. Specimens from each case were routinely stained with hematoxylin and eosin, GMS and PAS. Immunohistochemical stains for CD68 and CK-pan were also performed. Mouse monoclonal antibodies against human CK-pan and CD68 and MaxVision™/HRP against mouse and rabbit were used in these tests. All of the 18 patients were otherwise healthy apart from the disease, with no history of surgery or ocular trauma. The study was approved by the Ethics Committee of the Wenzhou Medical University Eye Hospital (approval number:2020–163-K-148).

**Table 1:** Demographic and clinical features of 18 patients with synthetic fiber conjunctival granulomas

Patient no.	Age range at last review, y	Duration	Location	Size, mm	Color	Symptoms
1	6 ~ 10	2 wk	RLL	1 × 2	White	FBS
2	1 ~ 5	1 y	LLL	10 × 5	Red-white	Lump, FBS
3	1 ~ 5	6 mo	RLL	7 × 6	White	Lump, FBS
4	6 ~ 10	1 y	LLL	10 × 2	Red-white	Lump, FBS
5	6 ~ 10	2 wk	RLL	3 × 8	Red-white	Lump, FBS, slight pain
6	16 ~ 20	1 y	RLL	4 × 1	Yellow-white	FBS
7	6 ~ 10	1 wk	RLL	3 × 2	Red-white	FBS
8	6 ~ 10	2 wk	LLL	4 × 6	Red	Lump, FBS
9	6 ~ 10	1 mo	LLL	2 × 3	Red	FBS, discharge
10	6 ~ 10	2 wk	RLL	3 × 6	White	FBS, itching, discharge
11	1 ~ 5	2 wk	LUL	2 × 7	White	Lump, FBS, discharge
12	16 ~ 20	1 wk	LLL	5 × 4	Red	FBS, slight pain, itching
13	21~	6 mo	RLL	2 × 4	Red-white	FBS, itching
14	1 ~ 5	2 mo	RLL	3 × 6	Red	FBS
15	6 ~ 10	3 wk	LLL	2 × 5	Red	FBS, slight pain, discharge
16	6 ~ 10	7 mo	RLL	3 × 5	Red-white	FBS
17	6 ~ 10	1 wk	LLL	5 × 7	White	Lump, FBS
18	11 ~ 15	1 mo	RLL	4 × 6	Red-white	FBS, itching

*LLL* Left lower lid, *LUL* Left upper lid, *RLL* Right lower lid, *FBS* Foreign body sensation, *mo* months, *y* years, *wk* weeks, *F* Female, *M* Male

## Results

**Fig. 1:**
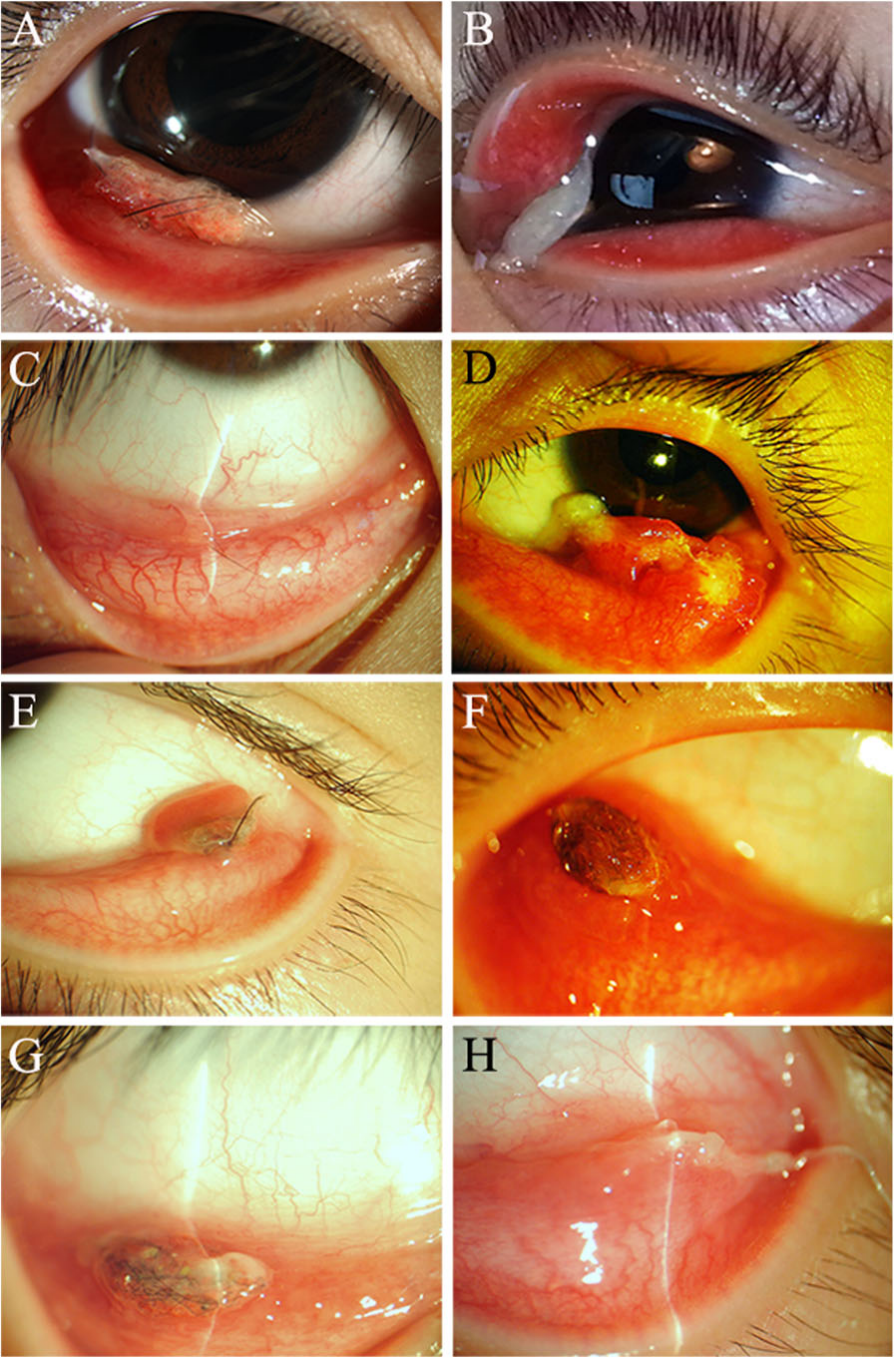
Different locations, sizes, and shapes of synthetic fiber conjunctival granulomas. **a** A granuloma present on the lower eyelid. **b** Conjunctival lesion located on the upper eyelid. **c** The smallest conjunctival granuloma seen in the inferior eyelid fornix of the right eye measuring 1 × 2 mm. **d** The largest lesion seen in the inferior eyelid fornix of the left eye measuring 5 × 10 mm. **e**-**h** Photographs show the various shapes of the lesions, such as round, oval, fabiform, or other irregular forms

A total of 18 patients with SFCG were included in this study. Males constituted 38.9% (7/18) and females 61.1% (11/18). The patients ages ranged from 3 to 31 years (9.3 ± 6.6 years). Among the patients’ granulomas, 2 masses were located in the inferior palpebral conjunctiva and 15 masses were located in the inferior fornix (Fig. 1a). Only one mass was located in the left upper eyelid (Fig. 1b). The unilateral eye was involved in all of the patients. The right eye was involved in 55.6% (10/18) and the left eye was involved in 44.4% (8/18). We collected all of the patients’ medical records. No previous history of cutaneous lesions or trauma was noted. Physical and ocular examinations revealed normal findings except for a soft, non-tender mass in the conjunctiva.

Essential information and clinical features of the 18 patients are summarized in Table 1. All of the patients reported foreign body sensation from 1 week up to 24 months prior to treatment. Other common symptoms included slight pain (3/18), itching (4/18), and eye discharge (4/18). The color and size of the conjunctival granulomas caused by synthetics fiber varied. These nodules ranged from 1 × 2 mm to 5 × 10 mm in size (Fig. 1c and d) and white to red in color. They appeared solid or cystic and did not adhere to the surrounding tissues (Table 1). The shapes of the granulomas were round, oval, fabiform, or other irregular forms. The most common shape was oval (Fig. 1e-h). Clinical examination revealed the lesions were non-palpable or discernible externally in most patients. Upon eversion of the eyelids, an elevated, well-defined, solitary nodule was found beneath the transparent palpebral conjunctiva with various synthetic fibers. The adjacent tissue was not inflamed or incrassated. External morphologies of the eyelashes were normal in all of the cases, and distichiasis, trichiasis, or ectopic cilia were not observed.

**Fig. 2:**
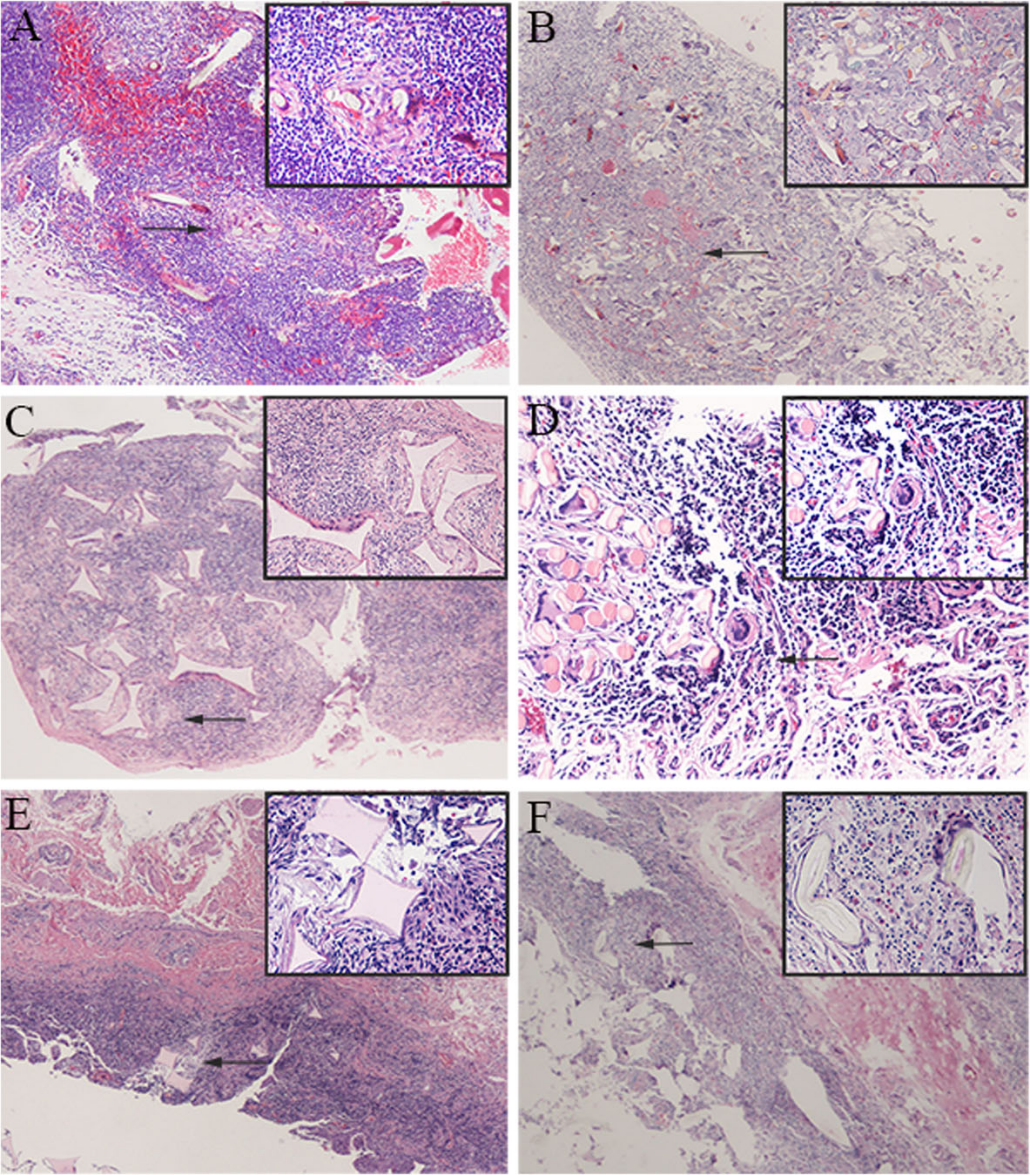
Histopathologic findings. **a** Photomicrograph showing the proliferation of granulation tissue with a large amount of inflammatory cell infiltration (hematoxylin-eosin, × 100; insets × 400). **b** Refractile foreign fibers noted in edematous connective tissue containing inflammatory cells (hematoxylin-eosin, × 40; insets × 400). **c**-**d** Synthetic fibers with relatively uniform size or central black granules (**c** hematoxylin–eosin, × 40; insets × 200. **d** hematoxylin–eosin, × 200; insets × 400). **e** The synthetic fibers are rhombic, pink, and surrounded by histiocytic giant cells (hematoxylin-eosin, × 40; insets × 400). **f** The synthetic fibers are pink, double spindle, translucent, and surrounded by histiocytic giant cells (hematoxylin-eosin, × 40; insets × 400)

**Fig. 3:**
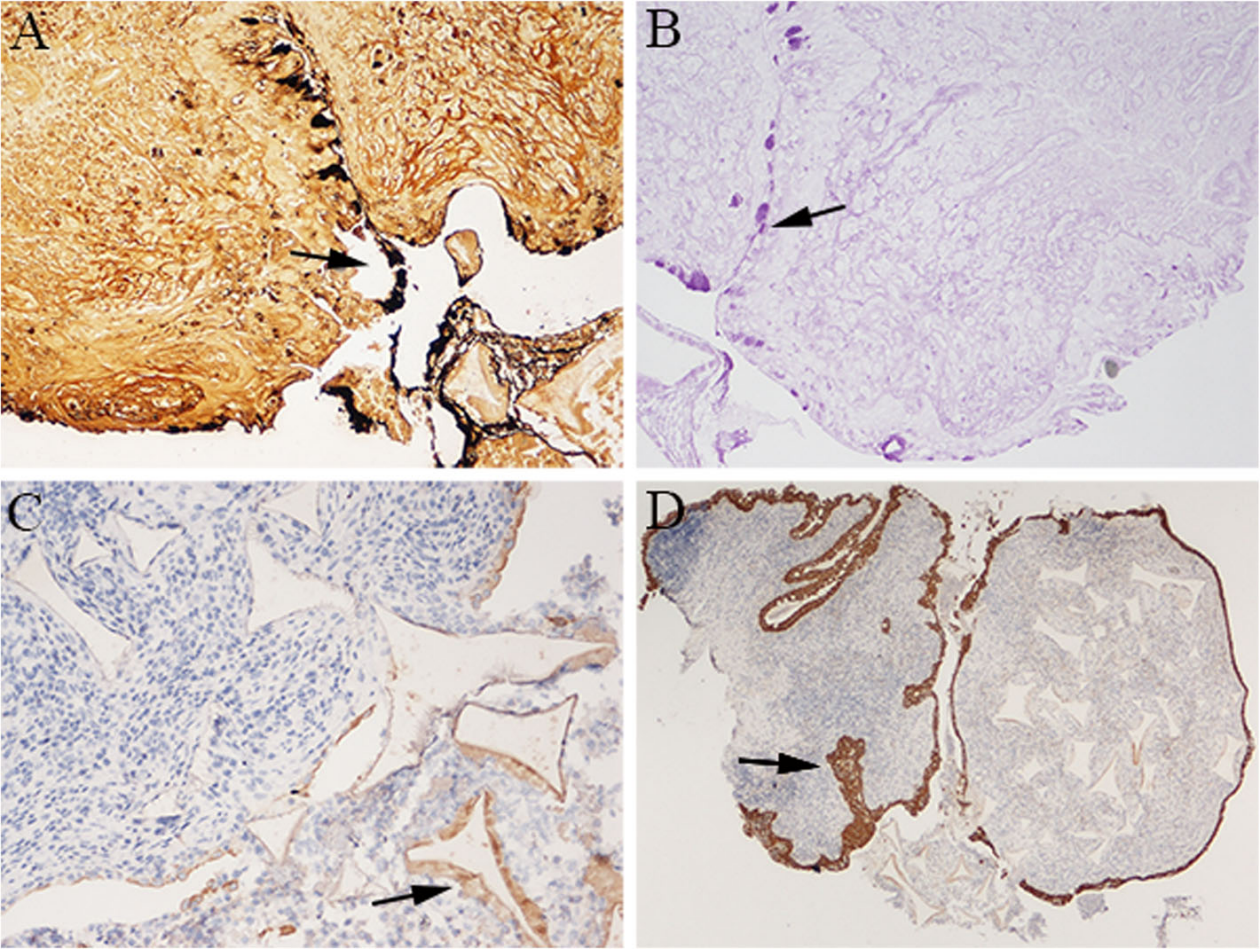
Immunohistochemical results. **a** GMS stain-positive goblet cells (magnification × 200). **b** PAS stain-positive goblet cells (magnification × 200). **c** CD68-positive macrophages (magnification × 200). **d** CK-pan positive conjunctival epithelial cells (magnification × 40)

Microscopic examination revealed a proliferation of inflammatory granulation tissue with many inflammatory cells including lymphocytes, plasma cells, eosinophils and neutrophils (Fig. 2a). Refractile foreign fibers were noted in edematous connective tissue containing neutrophils, foreign body giant cells and multinucleated histiocytes (Fig. 2b). Synthetic fibers were identified by their relatively uniform size, and some fibers exhibited central black granules (Fig. 2c and d). Most of the photomicrographs in our cases had similar pathological findings; however, case 2 had different sized mammillary-shaped red-white foreign bodies. Histological examination also revealed an inflammatory process in the substantia propria covered by conjunctival epithelium. Interestingly, we found some rhombic, pink substances (Fig. 2e) and a ring-like double spindle, translucent substance (Fig. 2f) that was not confirmed. Differential diagnoses should include non-infectious, immune, and infection-mediated conjunctival granulomas from the point of histopathology. Immunohistochemistry demonstrated that the goblet cells were positive for GMS stain and PAS stain (Fig. 3a and b) and the macrophages were positive for CD68 (Fig. 3c). The evaluation also showed positive staining with CK-pan (Fig. 3d). Most GMS stains, PAS stains, CK-pan and CD68 -positive cells were around the synthetic fibers of the granulation tissues.

**Fig. 4:**
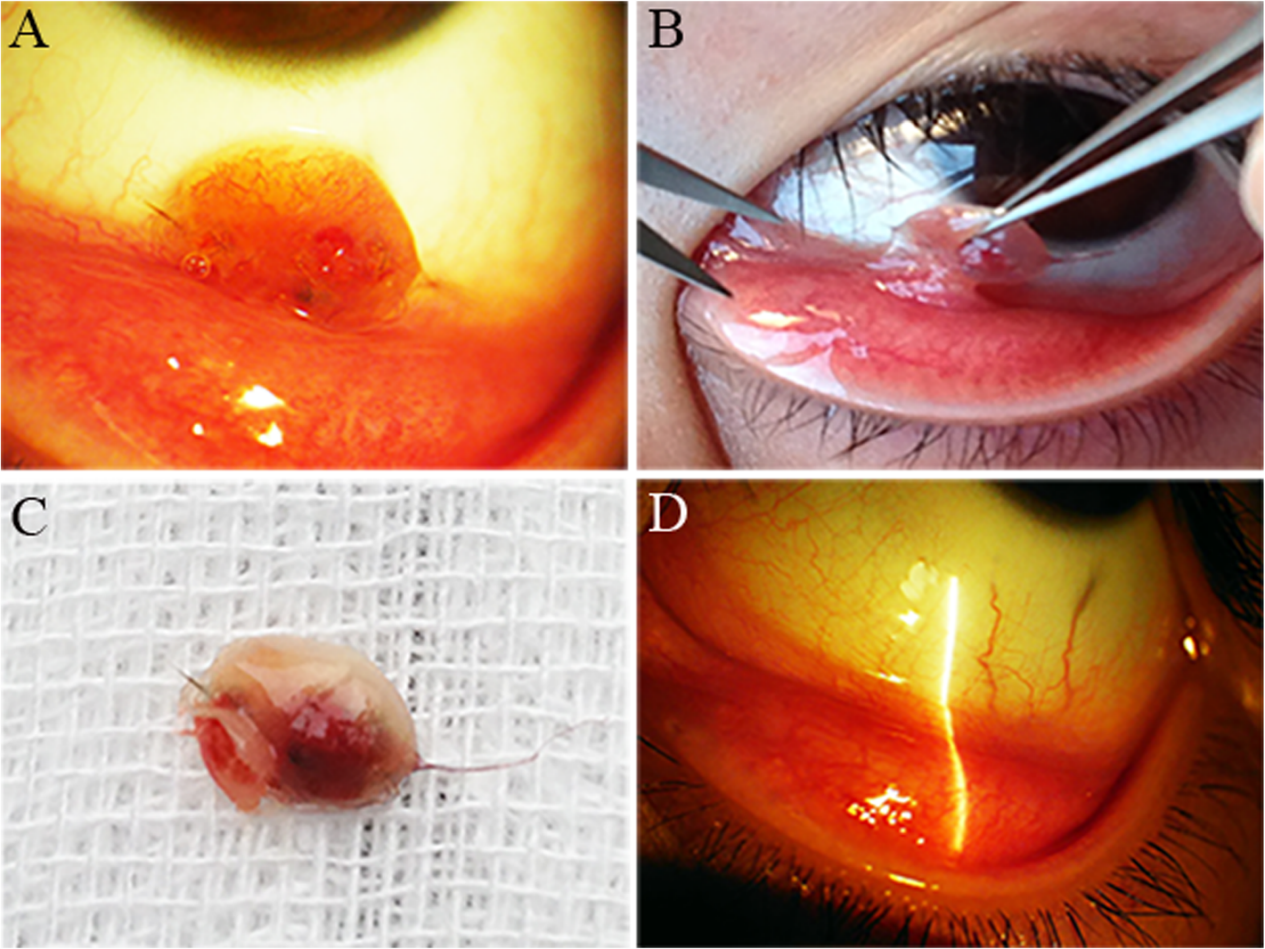
The surgical procedure of removing synthetic fiber conjunctival granulomas. **a** and **b** Photographs show the surgical procedure of cutting the lesion. **c** Photograph shows the mass cutted off from the eyelid. **d** Postoperative photograph shows no recurrence 10 months after surgery

Management modalities included surgical excision of the granulomas (Fig. 4a-c) and the administration of topical corticosteroid eye drops. The lesions completely resolved. Only 1 patient in the sample (5.6%) underwent general anesthesia due to his young age and anxiety, and the rest (94.4%) underwent topical anesthesia. All of the lesions completely resolved after treatment. During a mean follow-up period of 12 months (range, 8–20 months), none of patients showed significant recurrence or foreign body sensation after surgical debulking. There was no abnormality in their conjunctivas and corneas (Fig. 4d).

## Discussion

This study presents the largest series of patients with SFCG, improving the medical record data regarding this disease. The granuloma is a result of chronic inflammatory reaction. Synthetic fibers and hair exhibit good histocompatibility. Therefore, they will induce local chronic inflammation that is different from acute inflammatory reaction caused by bacteria when they embed in the underlying stroma and are encapsulated by mucous. Local chronic inflammation is usually asymptomatic. In addition, the mass mainly occurs in the inferior eyelid fornix [5]. Thus, patients rarely have ocular irritation symptoms, it is not easy to identify, and is often an occasional finding.

Prior studies have demonstrated a strong relationship between the lesions and various other objects containing synthetic fibers, including pullover sweaters, bedding, and blankets [3, 4, 7]. In our study, the etiology was identical to prior reports. Therefore, we believe avoiding close exposure to potential primary sources of synthetic fibers is crucial for preventing inflammatory granuloma growth.

SFCG was first reported by Weinberg et al. (1984) in a case series of 5 patients [1]. To date, 20 additional patients with conjunctival foreign body granulomas caused by synthetic fibers have been reported (PubMed database research, Table 2). As reported in these cases, the lesions were usually unilateral and mainly in the inferior fornix [3]. Compared to adolescents, children usually have a higher incidence of granulomas because they are more likely to be exposed to stuffed animals [8]. The ages of patients in the published cases ranged from 26 months to 17 years old [1, 2]. Based on the reviewed literature, including ours, the most common clinical symptom of SFCG is longstanding foreign body sensation; however, the majority of patients most often neglect the symptoms, which typically were present from many weeks up to a few months after exposure [3, 6, 7, 9]. Patients are usually referred to the hospital because of an occasional finding of a mass in the eye. It is usually recognized weeks or months after lesion formation. Of all the cases, only one patient reported by Farooq et al. presented with ocular irritation in her left eye [10]. They found the girl had developed severe keratitis and corneal ulceration. In our case series, in addition to foreign body sensations, other symptoms or signs including lumps and slight pain have been reported, and no other discomfort or ocular symptoms were demonstrated.

**Table 2:** Demographics and macroscopic findings in patients with synthetic fiber granulomas

Authors	Case	Age (years)	Gender	Duration	Location (conjunctival)	Size (mm)	Color
Weinberg JC et al. [1]	1	6	Male	2 months	Inferior fornix, OS	10 × 4 × 2	Pink
	2	8	Female	6 months	Inferior fornix, OS	7 × 2.5	Green
	3	16	Female	6 weeks	Superior fornix, OD	7 × 5 × 3	White, blue-black
	4	4	Male	2 months	Inferior fornix, OD	N/D	N/D
	5	17	Female	Several months	Inferior fornix, OD	4 × 4	N/D
Shields et al. [2]	6	2.2	Female	3 months	Inferior fornix, OD	7 × 7 × 5	Red-yellow
Arocker-Mettinger et al. [9]	7	2.6	Male	Several weeks	Inferior fornix, OS	N/D	Yellow-green
Resnick et al. [6]	8	5	Female	2 weeks	Inferior fornix, OD	4 × 3	Yellow-green
Offret et al. [11]	9	6	Female	N/D	Inferior fornix, OD	N/D	N/D
Ferry et a.l [7]	10	5	Female	N/D	Inferior fornix, OS	10 × 10	Yellow-white
Lueder et al. [12]	11	4	Female	1 month	Inferior fornix, OS	2 × 1	White
Yang et al. [8]	12	13	Female	N/D	Inferior fornix, OS	4 × 3	Blue
Enzenauer et al. [3]	13	4.5	Female	10 months	Inferior fornix, OS	15 × 10	White
Schmack et al. [4]	14	7	Female	5 months	Inferior fornix, OD	5 × 4 × 2	White-yellow
	15	2	Female	4 weeks	Inferior fornix, OD	10 × 4 × 3	Brown
Farooq et al. [10]	16	2	Female	3 weeks	OS	N/D	–
	17	5	Female	2 days	Inferior fornix, OS	7 × 5	N/D
Batta et al. [13]	18	3	Male	2 weeks	Inferior fornix, OD	10 × 2 × 3	Gray
Aliakbar-Navahi et al. [14]	19	6	Female	1 month	Inferior fornix, OD	5 × 5	Red
Mak et al. [5]	20	7	Female	1 month	Inferior fornix, OS	3.5 × 1.5	N/D

*N/D* No data available

Schmack et al. reported that the presence of a unilateral inferior conjunctival mass was the most reliable clinical sign indicating SFCG [4]. In contrast to clinical signs, the histologic features of SFCG are much more characteristic and diagnostic. Synthetic fibers can be confirmed by their relatively uniform size, strong birefringence under polarized light, and black granular spots. The characteristic histological features of large amounts of inflammatory cells including lymphocytes, plasma cells, eosinophils, and neutrophils presenting around the fibers supported the diagnosis of SFCG. Schmack et al. reported that the differential diagnosis of synthetic fiber conjunctival granuloma should include ophthalmia nodosa (insect and plant hairs), ligneous conjunctivitis, chalazion, pyogenic granuloma, dermoid, papillary hyperplasia, atypical dermoids or dermolipoma, or neoplasms such as rhabdomyosarcoma and vascular tumors [2, 4, 12] Many ophthalmologists may diagnose conjunctival masses as conjunctival dermoids because of their appearance. Although conjunctival dermoid presents as a mass with fine hairs adhering to the conjunctival fornix, the STR analysis could demonstrate that the inside hair is homologous with the mass, which is the primary difference between synthetic fiber conjunctival granuloma and conjunctival dermoid. The histopathologic and immunohistochemistry findings could also assist with correct diagnoses.

All of the microscopic examinations of our patients’ specimens revealed similar results, except in 2 cases. The foreign bodies found in these cases had not been described in previous literature. We could not clearly identify the foreign bodies but noted that they were surrounded by granulomatous inflammatory reactions. We suspect they were synthetic fibers that were not previously identified so further investigations are needed.

Treatment of SFCG involves surgical excision of the foreign body and granuloma followed by topical corticosteroids. None of the granulomas recurred at a mean follow-up period of 12 months. In our study, all of the patients underwent surgical excision under surface anesthesia except for one patient who underwent general anesthesia. Surface anesthesia has a number of potential advantages such as minimal bleeding and discomfort. It is currently well established from a variety of studies that if the patient is young, anxious, or non-compliant and the lesion is present for a long period before being recognized or is deeply embedded, general anesthesia is often needed [3, 12].

However, this retrospective observational case series has a few inherent limitations. The data were collected over a 4-year period based on the pathologic database, entailing some incomplete specimen data information. Furthermore, the small size of the dataset also meant that it was not possible to demonstrate all-sided features of SFCG. Our preliminary data still needs to be verified and enriched by investigations with more patients.

## Conclusions

The present study was designed for the further investigation of SFCG. In conclusion, the diagnosis of teddy bear granulomas can be challenging for ophthalmologists. SFCG may be more common than previously realized. As the number of accurate reports increases, these significant findings will help ophthalmologists develop a better understanding of this disease and improve awareness of these lesions. The diagnosis should be considered in any patient with a painless mass in the palpebral conjunctiva or conjunctival fornix, which are by far the most common locations. Definite diagnosis can be made via microscopic evaluation. The best treatment is surgical excision followed by topical corticosteroid eye drops, which is clinically proven to be effective. Avoiding potential primary sources of synthetic fibers should prevent the growth of conjunctival granuloma. Hence, it could conceivably be hypothesized that early diagnosis and active and effective treatment will be possible. This study’s results can provide a clinical basis for prevention and treatment of this disease.

## Supplementary Information

**Additional file 1.** Clinical photographs of 18 patients with synthetic fiber conjunctival granuloma (**only for review)**.

## Data Availability

The datasets used and/or analysed during the current study available from the corresponding author on reasonable request.

## Abbreviation

SFCG: Synthetic fiber conjunctival granuloma
