# Supplementary material for: Clinicopathologic characteristics and surgical outcome of synthetic fiber conjunctival granuloma
Source: BMC Ophthalmol. 2020 Nov 16;20:448. doi: 10.1186/s12886-020-01717-1 (PMC7670615; doi:10.1186/s12886-020-01717-1)

Clinical photographs of 18 patients with synthetic fiber conjunctival granuloma

Case 1

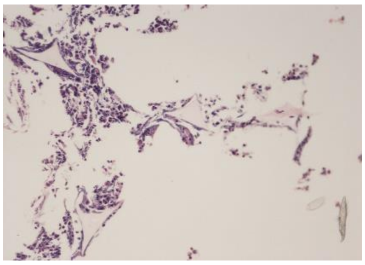

Case 2

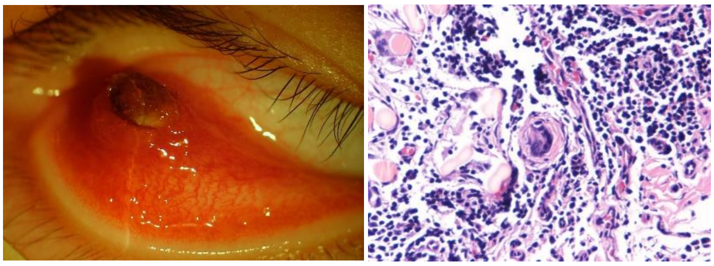

Case 3

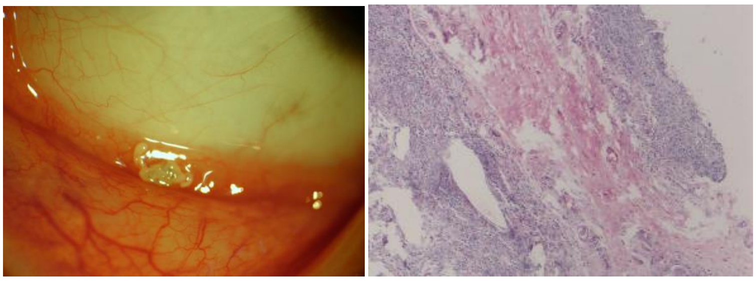

Case 4

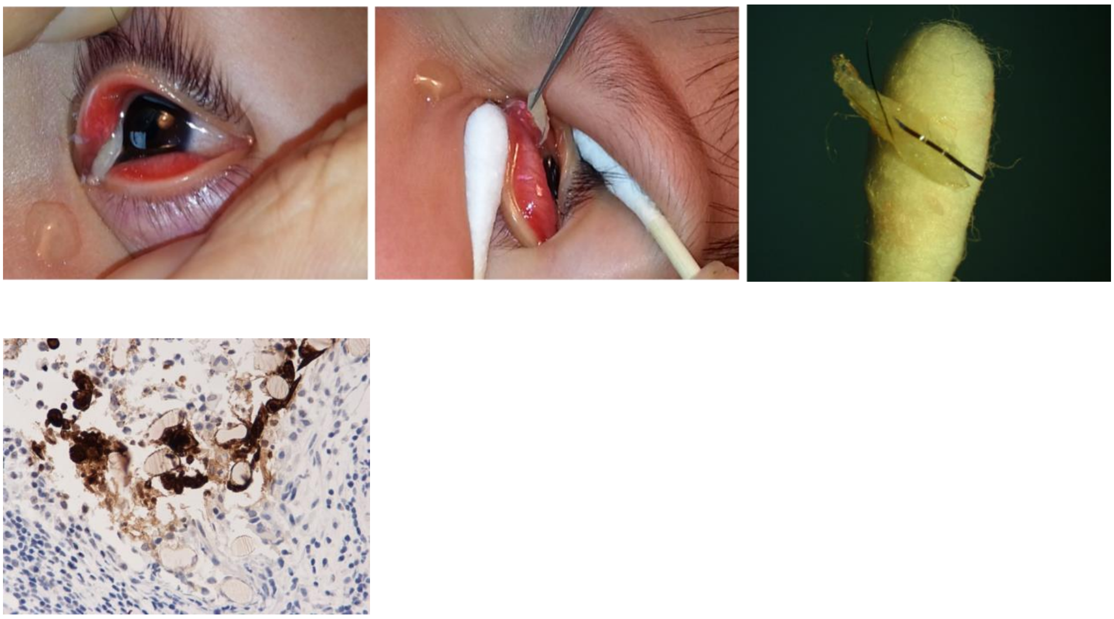

Case 5

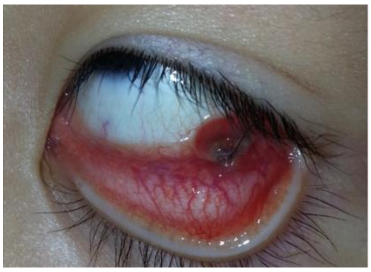
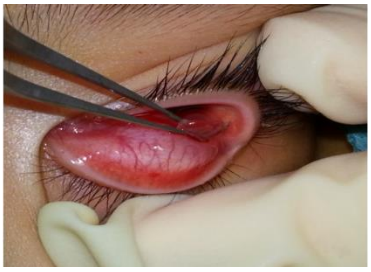
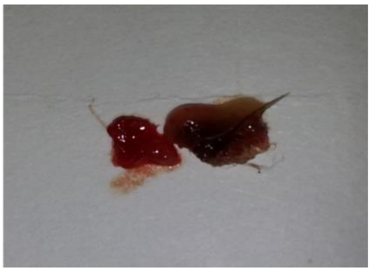
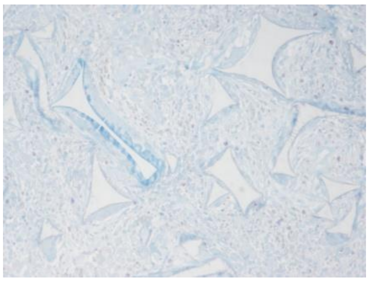

Case 6

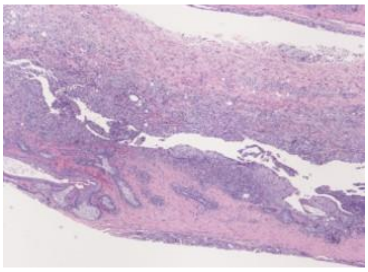

Case 7

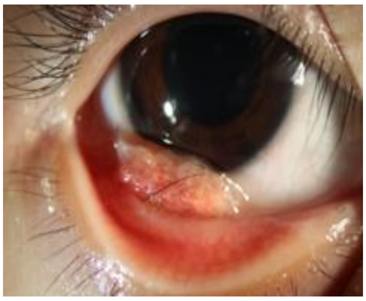
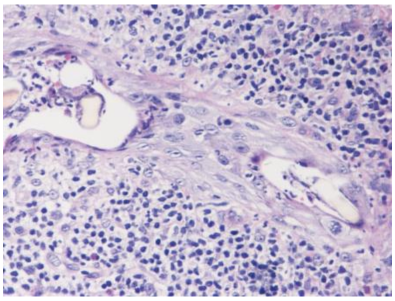

Case 8

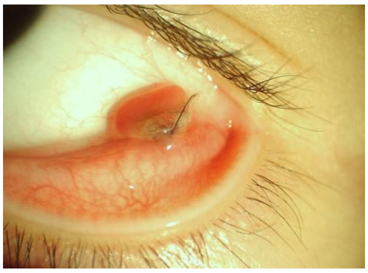
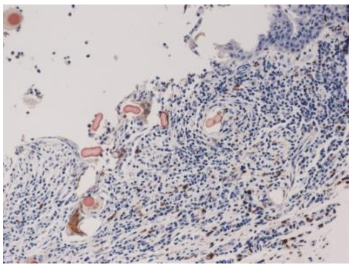

Case 9

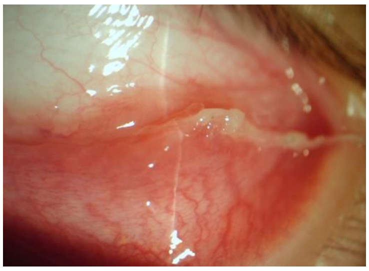

Case 10

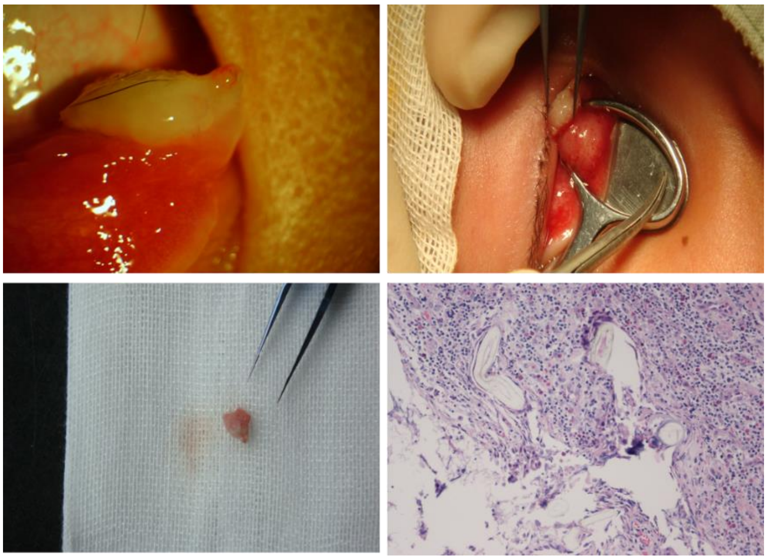

Case 11

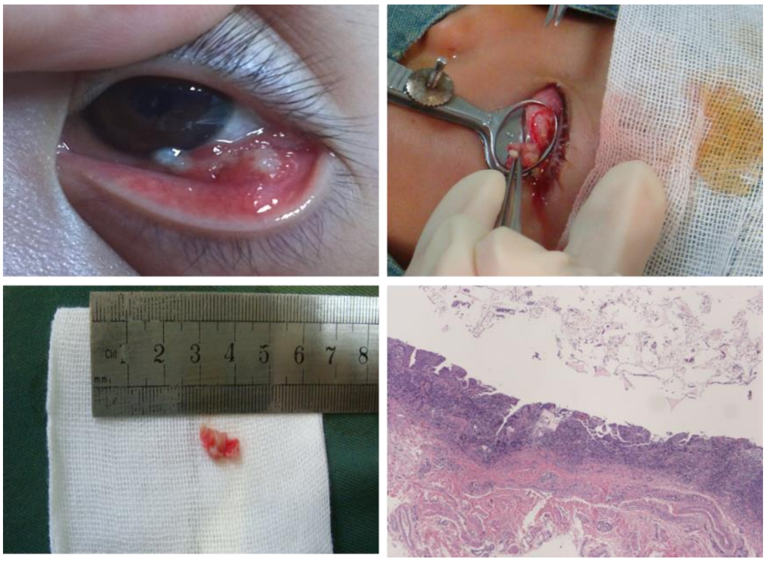

Case 12

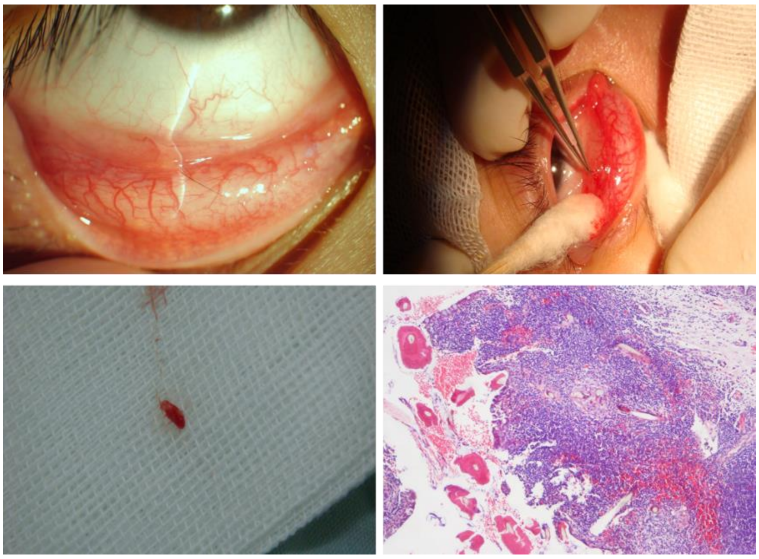

Case 13

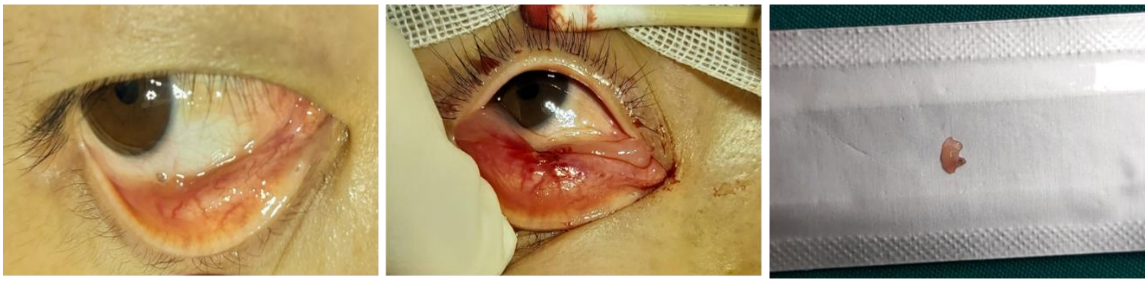

Case 14

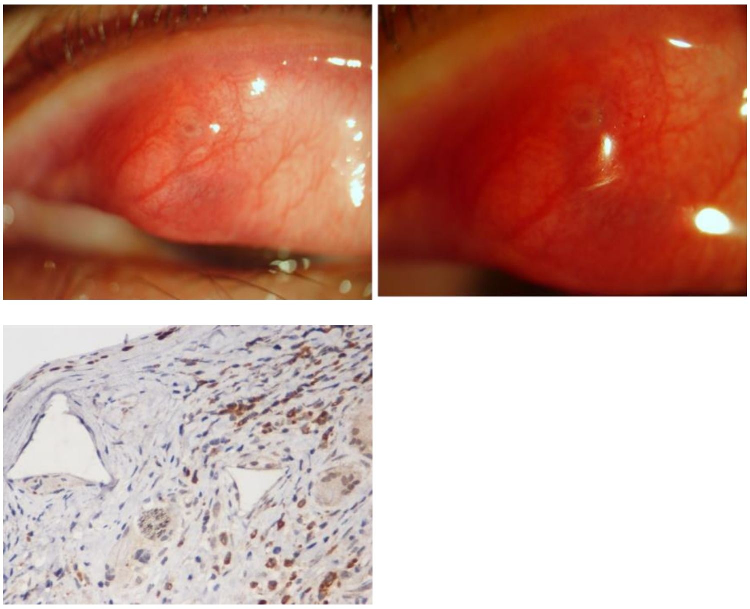

Case 15

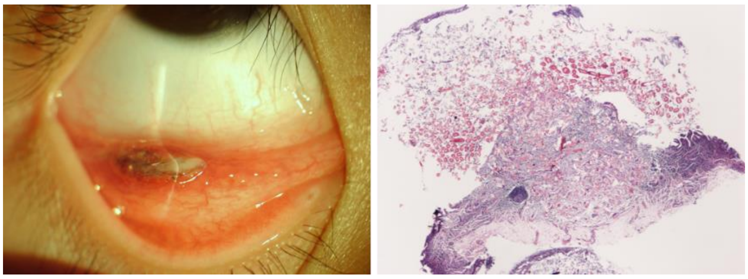

Case 16

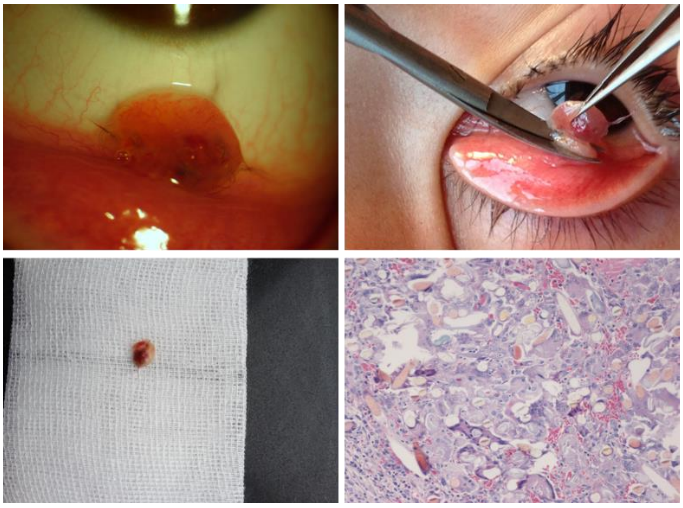

Case 17

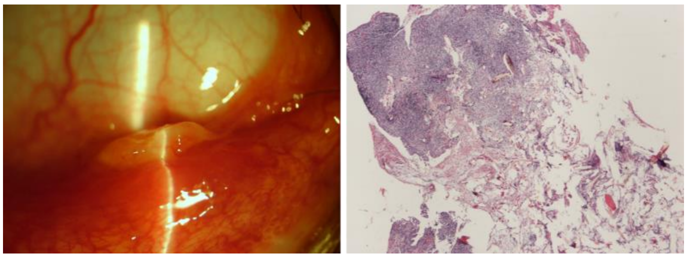

Case 18

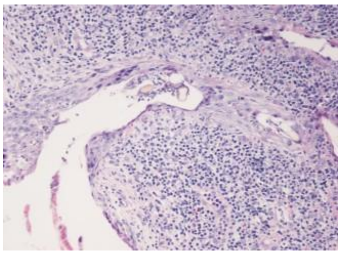

Supplement: Supplementary file 1 — Additional file 1. Clinical photographs of 18 patients with synthetic fiber conjunctival granuloma (only for review). [file 12886_2020_1717_MOESM1_ESM.pdf]
